# Self-reported physical functioning and physical fitness in glioma patients

**DOI:** 10.1093/nop/npaf076

**Published:** 2025-07-31

**Authors:** Marieke E C Blom, Maxine Gorter, Vera Belgers, Jantine G Röttgering, Philip C de Witt Hamer, Johanna M Niers, Marike R van Lingen, Mona L M Zimmermann, Hans Knoop, Martin Klein, Linda Douw

**Affiliations:** Cancer Center Amsterdam, Brain Tumor Center, Amsterdam, The Netherlands; Amsterdam UMC Location Vrije Universiteit Amsterdam, Anatomy and Neurosciences, Amsterdam, The Netherlands; Cancer Center Amsterdam, Brain Tumor Center, Amsterdam, The Netherlands; Amsterdam UMC Location Vrije Universiteit Amsterdam, Anatomy and Neurosciences, Amsterdam, The Netherlands; Amsterdam UMC Location Vrije Universiteit Amsterdam, Neurology, Amsterdam, The Netherlands; Cancer Center Amsterdam, Brain Tumor Center, Amsterdam, The Netherlands; Amsterdam UMC Location Vrije Universiteit Amsterdam, Medical Psychology, Amsterdam, The Netherlands; Cancer Center Amsterdam, Brain Tumor Center, Amsterdam, The Netherlands; Amsterdam UMC Location Vrije Universiteit Amsterdam, Neurosurgery, Amsterdam, The Netherlands; Cancer Center Amsterdam, Brain Tumor Center, Amsterdam, The Netherlands; Amsterdam UMC Location Vrije Universiteit Amsterdam, Neurology, Amsterdam, The Netherlands; Cancer Center Amsterdam, Brain Tumor Center, Amsterdam, The Netherlands; Amsterdam Neuroscience, Amsterdam UMC Location Vrije Universiteit Amsterdam, Amsterdam, The Netherlands; Cancer Center Amsterdam, Brain Tumor Center, Amsterdam, The Netherlands; Amsterdam UMC Location Vrije Universiteit Amsterdam, Anatomy and Neurosciences, Amsterdam, The Netherlands; Amsterdam Neuroscience, Amsterdam UMC Location Vrije Universiteit Amsterdam, Amsterdam, The Netherlands; Cancer Center Amsterdam, Brain Tumor Center, Amsterdam, The Netherlands; Amsterdam UMC Location Vrije Universiteit Amsterdam, Anatomy and Neurosciences, Amsterdam, The Netherlands; Amsterdam UMC Location University of Amsterdam, Medical Psychology, Amsterdam, The Netherlands; Amsterdam UMC Location Vrije Universiteit Amsterdam, Medical Psychology, Amsterdam, The Netherlands; Cancer Center Amsterdam, Brain Tumor Center, Amsterdam, The Netherlands; Amsterdam Neuroscience, Amsterdam UMC Location Vrije Universiteit Amsterdam, Amsterdam, The Netherlands; Cancer Center Amsterdam, Brain Tumor Center, Amsterdam, The Netherlands; Amsterdam UMC Location Vrije Universiteit Amsterdam, Anatomy and Neurosciences, Amsterdam, The Netherlands

**Keywords:** brain tumors, glioma, physical fitness, physical functioning

## Abstract

**Background:**

Knowledge about glioma patients’ physical functioning and physical fitness throughout the disease trajectory is limited. We analyzed self-reported functioning and fitness in a large sample of glioma patients preoperatively and after primary treatment.

**Methods:**

We used the physical functioning subscale of the 36-Item Short Form Health Survey (SF36) and three questions about physical fitness from the Checklist of Individual Strength (CIS20). Scores were compared to age-, sex-, and education-matched population controls, preoperatively and after treatment. In patients with repeated assessments, changes over time were analyzed. Correlations between patient, disease, and treatment characteristics and functioning and fitness at both time points, and with change over time, were explored. Analyses were performed separately for World Health Organization grade II, III, and IV glioma.

**Results:**

Grade III patients had significantly lower functioning than controls, both preoperatively and after treatment, and declined over time. No significant differences with controls were found for grade II and IV patients, or for fitness. Grade II patients reported better functioning than grade III and IV preoperatively and better than grade III after treatment. Lower Karnofsky Performance Status was generally related to lower functioning and fitness, while older age, female sex, and lower education appeared in subgroup analyses. Age, neurological disabilities and tumor histology were associated with changes over time.

**Conclusions:**

Grade III glioma patients showed poorer functioning than population controls, which declined over time. Grade II patients reported better outcomes than the other subgroups, although individual variability was high. These findings highlight the need for personalized approaches addressing functioning and fitness in glioma.

**Key Points**

- Grade III glioma patients show poorer physical functioning than controls.
- There is large individual variation in functioning and fitness across tumor grades.
- Personalized care is needed to address functioning and fitness in glioma.

**Importance of the Study**

Glioma patients often experience a variety of symptoms throughout their disease trajectory, with reduced physical functioning and physical fitness being common. However, the levels and course of self-reported functioning and fitness are poorly understood. This study identified particularly low functioning in grade III glioma patients both preoperatively and after primary treatment, compared to the general population. Although grade II patients reported better outcomes than the higher tumor grade patients, the considerable individual variability across all tumor grades underscores the need for personalized care. While commonly expected correlates, such as age and neurological disabilities, did not consistently emerge, KPS was a reliable correlate of functioning and fitness. Our findings highlight the value of broadening our perspective on quality of life and addressing potential limitations in functioning and fitness in glioma patients. We underscore the need for personalized strategies to improve these aspects of health.

Gliomas are the most common primary brain tumors. The diagnosis of glioma implies a poor prognosis for virtually all patients. Depending on tumor characteristics, treatment consists of surgery followed by radiotherapy and/or chemotherapy, followed by a varying period of stable disease, after which most tumors recur.^1^ Patients often experience multiple symptoms whose presence and burden may vary during the course of the disease.^2–4^

Glioma patients commonly experience reduced physical functioning and fitness, alongside symptoms such as fatigue, cognitive deficits, and emotional distress.^5,6^ Physical functioning impairments can manifest as difficulty in performing activities of daily living, while reduced aerobic functioning is most often reflected by lower levels of physical fitness (*Supplementary Figure 1*).^7^ Both functioning and fitness can be measured by either subjective or objective measurements. Questionnaires, such as patient-reported outcome measures (PROMs), can be used as subjective measurements. As an example, the 6-minute walk test (6MWT) can be used to objectively measure physical functioning. Objectively measured physical fitness encompasses the functioning of the heart, lungs, and muscles during physical activity, expressed as the maximum oxygen uptake (VO_2_max), and can be assessed with a cardiopulmonary exercise test (CPET).^7^ Functioning and fitness are related concepts, but focus on different aspects of physical health: functioning focuses more on the mechanical aspects of performing daily activities and exercise, while fitness relates best to cardiovascular health. The extent to which glioma patients experience reduced functioning or fitness, and when this occurs during the disease trajectory, is not well understood.

Remarkably low levels of functioning and fitness, as assessed with objective physical tests, have been reported in glioma patients. Two studies in patients with low- and high-grade gliomas found very low mean VO_2_max values, measured 10 to 40 days after surgery, compared to reference values.^8,9^ Although somewhat higher VO_2_max values were reported in low-grade glioma patients after at least 6 months of stable disease, these values were still below expected values based on age and sex.^10,11^ At the same time, very little is known about self-reported fitness in these patients. An explorative study asked patients to rate how often they experienced reduced physical fitness in the past two weeks on a 7-point Linker Scale. The median response was 4, and it was one of the most commonly reported symptoms.^5^ It remains unclear whether glioma patients more often experience poor physical fitness than the general population, and how this potential symptom fluctuates over time.

Physical functioning has been studied more extensively. Scores on the 6MWT, which objectively measures the distance covered within 6 minutes of walking, in patients with recurrent glioma were low.^12,13^ In glioma patients after surgery and before adjuvant therapy, 6MWT scores were comparable to those of community-dwelling adults aged 80 to 89 years old, while the sample was on average 40 years younger.^14^ On the subjective side, the 36-Item Short Form Health Survey (SF36) is an often-used questionnaire to measure quality of life, including self-reported physical functioning.^15,16^ In a large heterogeneous sample of brain tumor patients admitted for surgery, a mean functioning score of 69.28 out of 100 was reported.^17^ Healthy individuals generally score between 80 and 100 on this subscale, depending on age.^18^ Within this brain tumor sample, worse functioning was associated with age over 50 years and having a malignant tumor. In another sample, 16 high-grade glioma patients were followed for 16 months after diagnosis, and the SF36 physical functioning score was measured every 4 months. Functioning was lowest at the time of diagnosis, improved most after 4 months of follow-up, and remained almost stable after 16 months.^19^

To better understand the course of self-reported functioning and fitness during the disease trajectory and to define correlates of change therein, we analyzed a large longitudinal cohort of newly diagnosed glioma patients, conducting all analyses separately for patients with grades II, III, and IV tumors. These patients were able and willing to participate in assessments of their physical functioning and fitness, as well as a range of other measures, thus representing a relatively well-functioning subset of the population, particularly of grade IV glioma. The primary aim of this study was to compare the level of self-reported functioning and fitness in glioma patients preoperatively and after primary treatment (resection/biopsy with/without chemo- and/or radiotherapy) with the general population. We additionally compared functioning and fitness across grades. Secondly, we aimed to compare the level of self-reported functioning and fitness between both time points to observe changes over time. Finally, we examined potential clinical correlates of functioning and fitness preoperatively and after primary treatment, and correlates of the change in functioning and fitness between both time points in glioma patients.

## Methods

### Study Population and Procedures

In this retrospective analysis, we used prospectively collected data from glioma patients diagnosed between 2007 and 2023 at Amsterdam UMC. We used data from two different databases. The first part of the collected data was from patients who were scheduled for surgery and referred to the Department of Medical Psychology, as part of routine clinical care, for a number of assessments, including completion of the SF36 questionnaire and Checklist of Individual Strength (CIS20).^20^ Patients were subsequently asked to complete these questionnaires again during stable disease, which was on average 1 year after surgery, after primary treatment had ended. The second part of the collected data was from patients who were selected for inclusion into an observational study during multidisciplinary neuro-oncology meetings, and part of participation included completing SF36 and CIS20 questionnaires at one or more time points. In the current work, we included data from patients who completed questionnaires preoperatively, and/or at any timepoint after primary treatment, and without progression. Primary treatment encompassed surgery alone, or surgery followed by adjuvant treatment (chemotherapy, radiotherapy, or chemoradiation).

Information on age, sex, educational level, tumor histology, tumor location, tumor lateralization, tumor grade, surgery (biopsy or resection), adjuvant treatment (chemotherapy, radiotherapy, chemoradiation, or none), presence of epilepsy, usage of dexamethasone, Karnofsky Performance Status (KPS)^21^ and neurological deficits via the National Institutes of Health Stroke Scale (NIHSS)^22^ were collected from the medical records. Educational level was categorized into low (lower general education, and lower vocational education), middle (intermediate general education, intermediate vocational education, higher professional education), and high (higher general education, and university-level education).

As controls, we used SF36 data of the general Dutch population derived from a Netherlands Organization for Applied Scientific Research (TNO) study.^15^ A nationwide, population-based health status survey for the purposes of generating normative data was conducted in 1996 and included 1742 subjects.^15^ We additionally selected CIS20 data of a large sample (*n* = 2288) from the general Dutch population derived from CentERdata, a research institute at Tilburg University in the Netherlands.^23^ CentERdata had access to a large panel used for surveys, which reflects the distribution of the Dutch population in age, sex, education level, and social and economic status.^20^ We matched each patient with two population controls based on age, sex, and educational level. The maximum allowed age difference between matched cases was 5 years, and cases were matched based on the same educational level (i.e., low, middle, or high) and sex.

### Self-reported Physical Functioning and Physical Fitness

Physical functioning was assessed with the Dutch version of the SF36.^15,16^ This questionnaire consists of 36 items that represent different multi-item scales. In this study, we only used the physical functioning subscale. This subscale consists of 10 questions measuring the ability to perform daily physical activities (eg, climbing several flights of stairs) with scores ranging from 0 to 100. A higher score means better self-reported physical functioning.

Physical fitness was assessed with three questions from the CIS20, which in total consist of 20 questions measuring fatigue.^20^ The three questions we selected were all focused on physical fitness, namely “I feel fit,” “I feel in poor condition physically,” and “I feel in excellent condition physically.” These questions were scored on a 7-point Linkert scale. We rescaled the second question so that higher scores (range 3–21) indicated better self-reported physical fitness.

### Statistical Analysis

All analyses were performed using R, version 4.2.3. Statistical significance was set at *P* < .05. These analyses were preregistered before performing the analyses (https://osf.io/uk3j5). We performed all analyses for grades II, III, and IV glioma patients separately.

To compare patients to controls, we used non-parametric Mann-Whitney U-tests after checking the normality of the outcome data. Additionally, we compared functioning and fitness across tumor grade using Kruskall–Wallis tests, followed by post hoc pairwise Wilcoxon tests with Benjamini–Hochberg corrections. To compare the level of self-reported functioning and fitness between timepoints within the subset of patients with longitudinal data available at both time points, we used Wilcoxon signed-rank tests, again after checking the normality of the outcome data. In addition, we calculated the difference in functioning and fitness scores between both time points in this subset. A positive score indicated an improvement, a negative score indicated a decline, and a score of zero represented no change.

To explore clinical correlates, we first did univariate analyses for all potential clinical correlates. We tested correlations between the level of functioning and fitness with the following patient, disease, and treatment characteristics: age, sex, educational level, tumor histology, tumor location, tumor lateralization, tumor grade, adjuvant treatment (chemotherapy, radiotherapy, chemoradiation, or none), presence of epilepsy, usage of dexamethasone, KPS and the NIHSS score. We calculated Spearman’s correlation coefficients for continuous variables, performed Mann-Whitney U-tests for dichotomous variables, and used Kruskal–Wallis tests for categorical variables. Significant characteristics, along with age, sex, and educational level, which we considered essential to include, were subsequently analyzed in a multiple regression analysis to investigate correlations among multiple variables. Collinearity was checked. In case of collinearity, the variables with the highest correlation with the dependent variable were chosen.

In addition, we explored potential correlates of change in functioning and fitness in the longitudinal subset of patients. First, we conducted univariate analyses with all patient, disease, and treatment characteristics. Subsequently, significant variables along with age, sex, and educational level were included in a linear mixed model to investigate correlations among multiple variables, after checking for collinearity.

Due to the limited number of grade IV patients with data available after primary treatment, we did not perform longitudinal comparisons and correlational analyses at the after primary treatment timepoint in this group.

## Results

### Sample Characteristics

In total, we analyzed data from 260 glioma patients preoperatively and from 109 glioma patients after primary treatment. Notably, not all patients with data after primary treatment also had data preoperatively, and vice versa. Specifically, 79 glioma patients had longitudinal data at both time points. Table 1 provides an overview of the number of glioma patients per tumor grade (II, III, and IV), including relevant patient characteristics. To include two controls per glioma patient preoperatively and after primary treatment, we selected twice as many controls.

**Table 1.** Patient Characteristics of Grade II, III, and IV Glioma Patients

Characteristic	Preoperatively			After primary treatment			Longitudinal		
Tumor grade	II	III	IV	II	III	IV	II	III	IV
N	115	60	85	70	31	8	50	23	6
Age, mean (SD), years	41.0 (12.5)	44.0 (13.2)	58.1 (12.7)	41.0 (11.0)	44.5 (10.9)	57.5 (14.8)	41.3 (10.8)	44.2 (11.6)	60.7 (14.6)
Sex, *n* (%)									
Male	67 (58.3)	31 (51.7)	65 (76.5)	44 (62.9)	15 (48.4)	6 (75.0)	31 (62.0)	11 (47.8)	4 (66.7)
Female	48 (41.7)	29 (48.3)	20 (23.5)	26 (37.1)	16 (51.6)	2 (25.0)	19 (38.0)	12 (52.2)	2 (33.3)
Educational level, *n* (%)									
Low (1-2)	12 (10.4)	3 (5.00)	12 (14.1)	9 (12.9)	1 (3.23)	2 (25.0)	6 (12.0)	1 (4.35)	2 (33.3)
Middle (3-5)	49 (42.6)	29 (48.3)	28 (32.9)	27 (38.6)	14 (45.2)	3 (37.5)	20 (40.0)	12 (52.2)	2 (33.3)
High (6-8)	52 (45.2)	28 (46.7)	44 (51.8)	34 (48.6)	16 (51.6)	3 (37.5)	24 (48.0)	10 (43.5)	2 (33.3)
Missing	2 (1.74)		1 (1.18)						
KPS, *n* (%)									
≤ 70	8 (6.96)	12 (20.0)	8 (9.41)	5 (7.14)	6 (19.4)	1 (12.5)	5 (10.0)	3 (13.0)	1 (16.7)
≥ 80	102 (88.7)	47 (78.3)	73 (85.9)	63 (90.0)	24 (77.4)	5 (62.5)	45 (90.0)	19 (82.6)	4 (66.7)
Missing	5 (4.35)	1 (1.67)	4 (4.71)	2 (2.86)	1 (3.23)	2 (25.0)		1 (4.35)	1 (16.7)
Tumor histology, *n* (%)									
Astrocytoma	65 (56.5)	23 (38.3)	5 (5.88)	40 (57.1)	7 (22.6)		29 (58.0)	6 (26.1)	
Oligodendroglioma	50 (43.5)	37 (61.7)		30 (42.9)	24 (77.4)		21 (42.0)	17 (73.9)	
Glioblastoma			80 (94.1)			8 (100)			6 (100)
Tumor lateralization, *n* (%)									
Left	65 (56.5)	31 (51.7)	48 (80.0)	38 (54.3)	20 (64.5)	4 (50.0)	29 (58.0)	14 (60.9)	3 (50.0)
Right	48 (41.7)	28 (46.7)	34 (40.0)	30 (42.9)	11 (35.5)	4 (50.0)	21 (42.0)	9 (39.1)	3 (50.0)
Both	2 (1.74)	1 (1.67)	3 (3.53)	2 (2.86)					
Tumor location, *n* (%)									
Frontal	48 (41.7)	34 (56.7)	35 (41.2)	27 (38.6)	18 (58.1)	4 (50.0)	15 (30.0)	12 (52.2)	3 (50.0)
Non-frontal	67 (58.3)	26 (43.3)	50 (58.8)	43 (61.4)	13 (41.9)	4 (50.0)	35 (70.0)	11 (47.8)	3 (50.0)
Epilepsy, *n* (%)									
Yes	94 (81.7)	49 (81.7)	60 (70.6)	57 (81.4)	28 (90.3)	7 (87.5)	43 (86.0)	22 (95.7)	5 (83.3)
No	21 (18.3)	10 (16.7)	24 (28.2)	13 (18.6)	3 (9.68)	1 (12.5)	7 (14.0)	1 (4.35)	1 (16.7)
Missing		1 (1.67)	1 (1.18)						
Type of surgery, *n* (%)									
Resection	110 (95.7)	57 (95.0)	81 (95.3)	68 (97.1)	31 (100)	8 (100.0)	50 (100)	23 (100)	6 (100)
Biopsy	5 (4.35)	3 (5.00)	4 (4.71)	2 (2.86)					
Chemo- and/or radiotherapy, *n* (%)									
Only radiotherapy				5 (7.14)	4 (12.9)	1 (12.5)	2 (4.00)	2 (8.70)	
Only chemotherapy				3 (4.29)			3 (6.00)		
Both				12 (17.1)	21 (67.7)	7 (87.5)	8 (16.0)	18 (78.3)	6 (100)
Only surgery				50 (71.4)	6 (19.4)		37 (74.0)	3 (13.0)	
Use of dexamethasone, *n* (%)									
Yes	5 (4.35)	9 (15.0)	34 (40.0)	4 (5.71)	2 (6.45)	3 (37.5)	1 (2.00)	2 (8.70)	2 (33.3)
No	107 (93.0)	49 (81.7)	47 (55.3)	64 (91.4)	27 (87.1)	5 (62.5)	46 (92.0)	20 (87.0)	4 (66.7)
Missing	3 (2.07)	2 (3.33)	4 (4.71)	2 (2.86)	2 (6.45)		3 (6.00)	1 (4.35)	
NIHSS, *n* (%)									
No neurological disabilities	90 (78.3)	31 (51.7)	22 (25.9)	45 (64.3)	11 (35.5)	2 (25.0)	33 (66.0)	8 (34.8)	2 (33.3)
One or more neurological disabilities	5 (4.35)	10 (16.7)	22 (25.9)	8 (11.4)	8 (25.8)	4 (50.0)	7 (14.0)	7 (30.4)	4 (66.7)
Missing	20 (17.4)	19 (31.7)	41 (48.2)	17 (24.3)	12 (38.7)	2 (25.0)	10 (20.0)	8 (34.8)	

KPS, Karnofsky Performance Score; NIHSS, National Institutes of Health Stroke Scale; SD, standard deviation.

Patient characteristics are at diagnosis for the longitudinal sample.

There was some missing data for these samples of glioma patients. For the SF36, data were missing preoperatively for 5 grade II, 2 grade III, and 2 grade IV patients, and after primary treatment for 1 grade II patient. In the longitudinal sample, SF36 data were missing for 1 grade II and 1 grade III patient. For the CIS20, preoperative data were missing for 3 grade II, 1 grade III, and 8 grade IV patients, and after primary treatment for 1 grade II and 1 grade III patient. In the longitudinal sample, CIS20 data were missing for 1 grade III and 2 grade III patients.

The median preoperative timepoint for the total sample of glioma patients was 12 days before surgery, with a range from 1 to 785 days (*Supplementary Figure 2*). The timepoint after primary treatment was a median of 421 days after surgery, with a range from 120 to 2154 days (*Supplementary Figure 3*).

### Physical Functioning and Physical Fitness of Grade II Patients Compared to Controls

**Figure 1.**
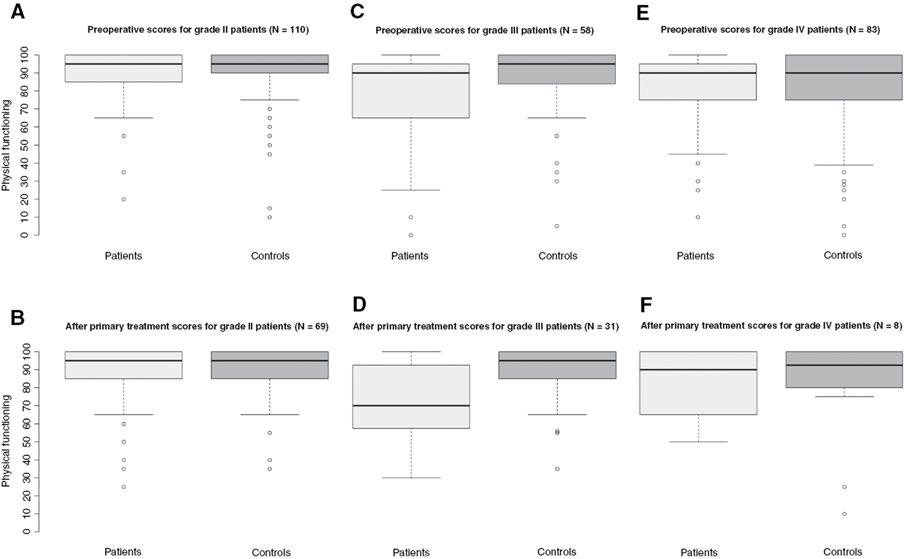
Physical functioning is shown preoperatively and after primary treatment for grade II (**A**, **B**), III (**C**, **D**), and IV (**E**, **F**) patients, respectively. A higher score means better physical functioning.

Preoperatively, there was no significant difference in functioning between grade II patients (median = 95, IQR = 86.25–100) and their matched controls (median = 95, IQR = 90–100; U = 11442, z = −0.81, *P* = .401; Figure 1A). Similarly, after primary treatment, functioning did not differ significantly between grade II patients (median = 95, IQR = 85–100) and controls (median = 95, IQR = 85–100; U = 4352, z = −1.01, *P* = .295; Figure 1B).

**Figure 2.**
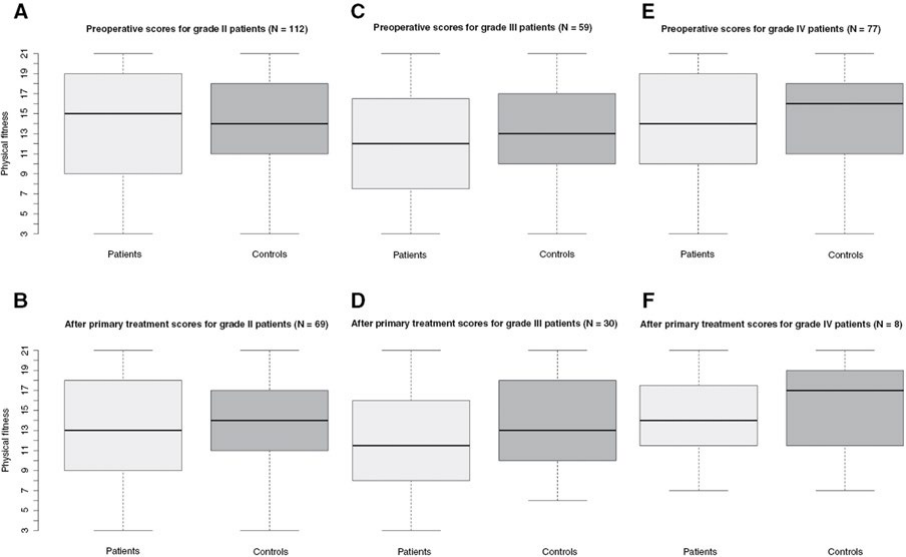
Physical fitness is shown preoperatively and after primary treatment for grade II (**A**, **B**), III (**C**, **D**), and IV (**E**, **F**) patients, respectively. A higher score means better physical fitness.

No significant differences were found in fitness either. Preoperatively, fitness scores were comparable between grade II patients (median = 15, IQR = 9–19) and controls (median = 14, IQR = 11–18; U = 12174, z = −0.44, *P* = .659; Figure 2A). After primary treatment, fitness also did not differ significantly between grade II patients (median = 13, IQR = 9–18) and controls (median = 14, IQR = 11.25–17; U = 4565.5, z = −0.48, *P* = .630; Figure 2B).

### Physical Functioning and Physical Fitness of Grade III Patients Compared to Controls

Preoperatively, functioning was significantly lower in grade III patients (median = 90, IQR = 66.25–95) compared to their controls (median = 95, IQR = 84.5–100; U = 2,508, z = –2.73, *P* = .005; Figure 1C). This difference remained significant after primary treatment, with grade III patients showing lower functioning (median = 70, IQR = 57.5–92.5) compared to controls (median = 95, IQR = 85–100; U = 507.5, z = –3.70, *P* < .001; Figure 1D).

In contrast, no significant differences were found in fitness. Preoperatively, fitness scores of grade III patients (median = 12, IQR = 7.5–16.5) were comparable to those of controls (median = 13, IQR = 10–17; U = 3,175.5, z = –0.95, *P* = .342; Figure 2C). Similarly, after primary treatment, fitness did not significantly differ between grade III patients (median = 11.5, IQR = 8–15.75) and controls (median = 13, IQR = 10–18; U = 710, z = -1.63, *P* < 0.104; Figure 2D).

### Physical Functioning and Physical Fitness of Grade IV Patients Compared to Controls

Preoperatively, functioning did not significantly differ between grade IV patients (median = 90, IQR = 75–95) and their matched controls (median = 90, IQR = 75–100; U = 6,168, z = –1.35, *P* = .173; Figure 1E). Similarly, after primary treatment, functioning was comparable between grade IV patients (median = 90, IQR = 67.5–100) and controls (median = 92.5, IQR = 80–100; U = 60.5, z = –0.21, *P* = .851; Figure 1F).

No significant differences were found in fitness either. Preoperatively, fitness scores of grade IV patients (median = 14, IQR = 10–19) did not differ significantly from those of controls (median = 16, IQR = 11–18; U = 5,555, z = –0.78, *P* = .434; Figure 2E). Similarly, after primary treatment, fitness did not significantly differ between grade IV patients (median = 14, IQR = 12.25–17.25) and controls (median = 17, IQR = 11.75–18.5; U = 54.5, z = −0.58, *P* = .579; Figure 2F).

### Physical Functioning and Physical Fitness Across Grade II, III, and IV Patients

A significant difference in preoperative functioning was found between tumor grades (H(2) = 18.2, *P* < .001). Post hoc comparisons showed that patients with grade II tumors had significantly better functioning than those with grade III (*P* = .001) and grade IV (*P* < .001). However, no significant difference was observed between patients with grade III and grade IV (*P* = .841). Regarding fitness, no significant differences were found between tumor grades preoperatively (H(2) = 2.53, *P* = .282).

After primary treatment, a significant difference in functioning across tumor grades was again found (H(2) = 11.3, *P* = .004). Post hoc comparisons revealed that patients with grade II tumors had significantly better functioning than those with grade III (*P* = .002). No significant differences were observed between grade II and IV (*P* = .499), nor between grade III and IV (*P* = .380). Similarly, no significant differences in fitness were found between tumor grades after primary treatment (H(2) = 1.95, *P* = .376).

### Longitudinal Change in Physical Functioning and Physical Fitness of Grade II Patients

**Figure 3.**
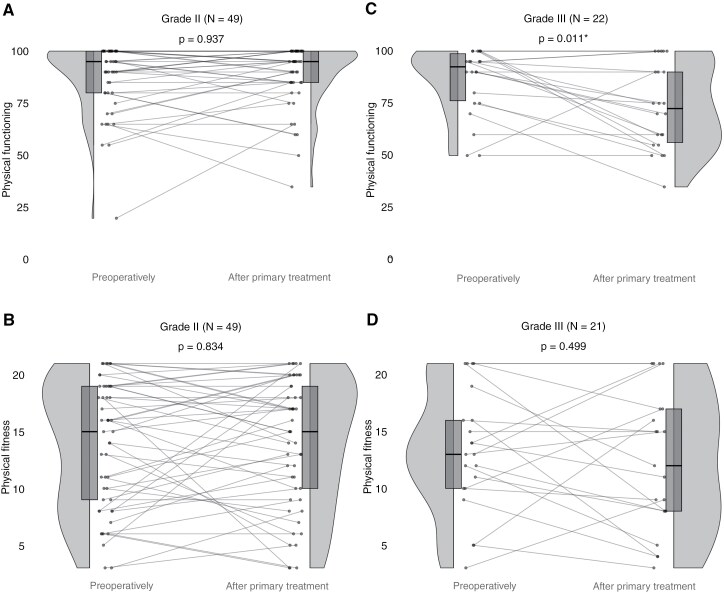
Longitudinal change for grade II patients in physical functioning (**A**) and physical fitness (**B**), and for grade III patients in physical functioning (**C**) and in physical fitness (**D**). A higher score means better physical functioning or physical fitness.

Functioning of grade II patients did not change significantly at the group-level (W = 327.5, z = −1.42, *P* = .937) between the preoperative timepoint (median = 95, IQR = 80–100) and after primary treatment (median = 95, IQR = 85–100) in the patients with longitudinal data available. However, there was large individual variability in change over time (Figure 3A). In this sample of 49 patients, 29% of the functioning scores declined by an average of 12.1 points, and 33% improved by an average of 14.7 points.

Similarly, fitness did not change at the group-level (W = 498.5, z = −0.57, *P* = .834) between the preoperative timepoint (median = 15, IQR = 9–19) and after primary treatment (median = 15, IQR = 10–19). Nonetheless, there was considerable individual variability in change over time (Figure 3B), with 43% of the fitness scores declining by on average 4.2 points, and 47% improving by on average 4.2 points.

### Longitudinal Change in Physical Functioning and Physical Fitness of Grade III

In grade III patients with longitudinal data, functioning was significantly lower after primary treatment (median = 72.5, IQR = 56.25–90) compared to the preoperative timepoint (median = 92.5, IQR = 76.25–98.75; W = 117.7, z = −0.15, *P* = .011; Figure 3C). Within this sample, 59% of patients experienced a decline in functioning, with an average decrease of 25.8 points, while 13.6% showed improvement, with an average increase of 16.7 points.

Fitness did not change significantly at the group level (W = 101.5, z = −0.24, *P* = .499) between the preoperative timepoint (median = 13, IQR = 10–16) and after primary treatment (median = 12, IQR = 8–17). However, individual variability was substantial (Figure 3D), as 57% of patients’ fitness scores declined by an average of 5.2 points, and 29% improved by an average of 6.8 points.

### Correlates of Physical Functioning and Physical Fitness

The results of the multivariate analyses examining potential correlates of functioning and fitness preoperatively for grades II, III, and IV, as well as after primary treatment for grades II and III, are presented in Tables 2 and 3 respectively. The results of the univariate analyses are presented in Supplementary Tables 1 and 2.

**Table 2.** Multivariate Analyses of Functioning and Fitness Preoperatively for Grade II, III and IV Glioma Patients

Characteristic	Standardized coefficients		Unstandardized coefficients		
SF36 physical functioning	β	B	Std. error	*t* value	*P* value
**Grade II**					
(Intercept)	−1.15	81.6	8.22	9.92	<.001***
Age	−0.18	−0.21	0.11	−1.92	.058
Sex	−0.11	−1.54	2.73	−0.57	.573
Educational level middle compared to low	0.36	5.19	5.04	1.03	.306
Educational level high compared to low	0.61	8.80	5.04	1.75	.084
KPS ≥ 80 compared to ≤ 70	0.80	11.5	5.61	2.05	.043*
**Grade III**					
(Intercept)	−1.59	34.9	20.2	1.73	.090
Age	0.04	0.08	0.23	0.33	.742
Sex	0.04	1.03	5.60	0.18	.854
Educational level middle compared to low	0.27	6.92	15.6	0.45	.658
Educational level high compared to low	0.55	13.8	15.9	0.87	.388
KPS ≥ 80 compared to ≤ 70	1.46	36.8	7.32	5.02	<.001***
**Grade IV**					
(Intercept)	−1.48	59.3	15.9	3.73	<.001***
Age	−0.07	−0.11	0.17	−0.66	.511
Sex	−0.21	−4.13	5.01	−0.83	.412
Educational level middle compared to low	0.59	11.4	6.77	1.69	.096
Educational level high compared to low	0.83	16.2	6.30	2.57	.012*
KPS ≥ 80 compared to ≤ 70	1.14	22.0	7.33	3.00	.004***
Tumor in right hemisphere compared to left	−0.34	−6.51	4.17	−1.56	.123
**CIS20 physical fitness**					
**Grade II**					
(Intercept)	−0.82	9.05	2.92	3.10	.003**
Age	−7e−04	−3e−04	0.04	−0.007	.994
Sex	−0.25	−1.36	1.04	−1.32	.192
Educational level middle compared to low	−0.04	−0.22	1.87	−0.12	.907
Educational level high compared to low	0.47	2.56	1.89	1.36	.178
KPS ≥ 80 compared to ≤ 70	0.78	4.26	2.07	2.06	.042*
**Grade III**					
(Intercept)	−1.83	−2.10	5.11	−0.41	.683
Age	0.18	0.09	0.06	1.46	.151
Sex	−0.22	−1.30	1.40	−0.93	.357
Educational level middle compared to low	0.99	5.77	3.92	1.47	.147
Educational level high compared to low	1.12	6.53	4.00	1.63	.109
KPS ≥ 80 compared to ≤ 70	1.17	6.84	1.80	3.80	<.001***
**Grade IV**					
(Intercept)	−1.34	4.64	4.72	0.98	.329
Age	0.06	0.03	0.05	0.52	.602
Sex	−0.10	−0.56	1.58	−0.36	.724
Educational level middle compared to low	0.32	1.77	2.11	0.84	.404
Educational level high compared to low	0.24	1.34	1.97	0.68	.499
KPS ≥ 80 compared to ≤ 70	1.24	6.92	2.22	3.12	.002**

*CIS20, Checklist Individual Strength; KPS, Karnofsky Performance Score; SF36, 36-Item Short Form Health Survey.*

* < .05, ** < .01, *** < .001.

**Table 3.** Multivariate Analyses of Functioning and Fitness after Primary Treatment for Grade II and III Glioma Patients

Characteristic	Standardized coefficients		Unstandardized coefficients		
SF36 physical functioning	β	B	Std. Error	*t* value	*P* value
**Grade II**					
(Intercept)	−1.69	74.0	10.8	6.86	<.001***
Age	−0.25	−0.38	0.17	−2.28	.026*
Sex	−2e−03	−0.04	3.94	−0.01	.992
Educational level middle compared to low	−0.01	−0.24	6.06	−0.04	.969
Educational level high compared to low	0.56	9.70	5.70	1.70	.094
KPS ≥ 80 compared to ≤ 70	1.54	26.5	7.34	3.61	<.001***
**Grade III**					
(Intercept)	−1.02	75.2	24.7	3.04	.006**
Age	−0.28	−0.54	0.34	−1.62	.118
Sex	−0.85	−17.8	7.11	−2.51	.019*
Educational level middle compared to low	0.92	19.5	18.9	1.03	.314
Educational level high compared to low	0.94	19.8	18.0	1.10	.282
KPS ≥ 80 compared to ≤ 70	0.71	15.0	8.76	1.71	.100
**CIS20 physical fitness**					
**Grade II**					
(Intercept)	−0.70	13.9	4.01	3.47	<.001***
Age	−0.23	−0.12	0.06	−1.92	.060
Sex	−0.06	−0.36	1.42	−0.25	.803
Educational level middle compared to low	−0.50	−2.86	2.33	−1.23	.225
Educational level high compared to low	0.07	0.38	2.20	0.17	.862
KPS ≥ 80 compared to ≤ 70	0.95	5.43	2.70	2.01	.048*
**Grade III**					
(Intercept)	−0.75	10.3	6.77	1.52	.142
Age	−0.12	−0.06	0.10	−0.60	.551
Sex	−0.44	−2.46	2.22	−1.11	.278
Educational level middle compared to low	0.86	4.82	6.17	0.78	.442
Educational level high compared to low	1.12	6.26	5.90	1.06	.299

*CIS20, Checklist Individual Strength; KPS, Karnofsky Performance Score; SF36, 36-Item Short Form Health Survey.*

* < .05, ** < .01, *** < .001.

Preoperatively, functioning was significantly correlated with KPS for grades II and III, while for grade IV, both KPS and educational level were significant predictors. Higher KPS scores and higher educational levels were associated with better functioning. After treatment, better functioning in grade II was linked to higher KPS and younger age, whereas in grade III, female sex was associated with poorer functioning. For fitness, preoperative KPS was a significant correlate across grades II, III, and IV, with higher KPS indicating better fitness. After primary treatment, KPS remained a significant predictor of fitness only for grade II.

Results of univariate analyses on changes in functioning and fitness over time are shown in Supplementary Table 3 (grade II) and Supplementary Table 4 (grade III). Multivariate analysis results are presented in Supplementary Table 5 (grade II) and Supplementary Table 6 (grade III). In grade II patients, those with neurological deficits reported better functioning at baseline but showed a greater decline over time. Regarding fitness, grade II patients with an oligodendroglioma reported lower fitness than those with an astrocytoma at baseline, yet their fitness improved more over time. Additionally, older patients showed smaller improvements in fitness over time. A similar pattern was observed for functioning in grade III patients.

### Sensitivity Analysis

We performed a sensitivity analysis comparing functioning and fitness with general population controls, including only patients whose assessments fell within a narrower time window between the date of surgery and the date of assessment. Specifically, we included patients with a preoperative assessment conducted up to 100 days before surgery (*Supplementary Figure 2*) and an after-primary-treatment assessment conducted between 280 and 550 days after surgery (*Supplementary Figure 3*), as most data fell within this time window and were considered clinically relevant. We found no differences compared to the primary analysis, showing the same pattern: functioning of grade III patients was significantly lower compared to controls, both preoperatively and after primary treatment (*Supplementary Table 7*).

## Discussion

This study aimed to better understand the course and correlates of self-reported physical functioning and physical fitness in grade II, III, and IV glioma patients. Grade III patients reported particularly poorer functioning compared to the general population, with a significant decline following primary treatment. Grade II patients showed higher functioning than grade III and IV patients. Lower KPS was generally linked to lower functioning and fitness, while older age, female sex and lower educational level were identified as correlates of lower functioning or fitness in particular subgroup or timepoint analyses. Age, neurological disabilities and tumor histology were associated with changes in functioning or fitness over time. The findings of this study underscore the importance of addressing functioning as one of the PROMs, especially for higher-grade glioma patients. Moreover, due to the considerable variability in changes in functioning and fitness over time, adopting a personalized approach is essential.

Our finding of poor self-reported functioning in grade III glioma patients is in line with the literature. Previous studies in high-grade glioma have reported low functioning preoperatively and also in the follow-up phase after treatment^19,24^ However, unlike our study, previous studies also reported significantly lower values of functioning in low-grade glioma patients after primary treatment compared to general population controls.^25^ In another study, patients with low-grade glioma and patients with meningioma had also significantly lower functioning after primary treatment compared to healthy controls.^26^ Clearly, functioning and fitness are topics of concern for glioma patients throughout the disease course. In the current study, patients were also compared to controls from the general population who may not have been considered healthy, as is often used in research, which means that we may have underestimated the difference between patients and controls.

We found no significant difference in functioning between grade IV patients and the general population controls. Higher tumor grade generally correlates with poorer prognosis,^27^ and we expected it would also correlate with lower functioning. In the overall glioma population, grade IV tumors are more common than grade II and III. However, we must underline that the included grade IV patients may not have been representative of the entire population of grade IV glioma patients, as they were physically and cognitively able and willing to fill out these questionnaires, which were part of a larger assessment of their functioning. Furthermore, the small number of grade IV patients available after primary treatment limited our ability to perform comprehensive analyses.

Additionally, we compared functioning and fitness between patients and matched controls based on age, sex, and educational level. Our results are likely influenced by age differences, as grade IV patients and their matched controls were notably older than those with grade II and III tumors. Since functioning typically declines with age,^28,29^ the difference between grade IV patients and their older controls may appear smaller, despite grade IV patients exhibiting lower absolute functioning compared to grade II patients.

In contrast to functioning, fitness did not significantly differ from controls. There is a difference between self-reported functioning and fitness: self-reported functioning assesses physical limitations, while self-reported fitness is more based on perceptions and expectations on cardiovascular health.^7^ The differences observed, also with regard to the correlates, are likely due to differences in the underlying concepts. However, this study does not allow us to determine the extent of overlap or distinction between functioning and fitness in this population.

We identified several factors that correlated with functioning and fitness preoperatively and/or after primary treatment in grade II, III, and IV patients. It is important to note that some subgroup sizes were small, and therefore, the results should be interpreted with caution. A clear correlate that emerged was the KPS. Higher KPS correlated with better functioning and fitness, except for grade III patients after primary treatment. KPS is a performance score that separates poorly functioning patients from those in normal condition, so we expected to find this correlation.^30^ In grade III patients, functioning correlated with sex after primary treatment. More specifically, females reported lower functioning compared to males. These findings align with previous research showing that female cancer patients often report more physical symptoms than males.^31^

We found a lower educational level to be correlated with lower functioning in grade IV patients preoperatively. Higher-educated individuals may be more physically active due to factors such as greater health awareness, healthier behaviors, better access to recreational facilities, and social norms prioritizing physical activity.^32,33^ Additionally, higher socioeconomic status, including educational attainment, is associated with increased physical activity levels in adults,^34^ contributing to better functioning and fitness. Notably, fewer patients with a lower educational level were included in this study, which may have introduced selection bias. Higher-educated individuals may be more likely to participate in research studies.

We found that older age was associated with lower functioning in grade II patients after primary treatment. This aligns with trends in the general population, where increasing age is linked to reduced physical outcomes, often due to declines in physical activity and muscle mass.^28,29^ However, this association was not consistently observed across other grades or at the other time point in our study. When examining changes over time, a significant interaction between age and time indicated that, in grade II patients, older patients showed smaller improvements in fitness. Similarly, in grade III patients, older age was associated with less improvement in functioning over time.

Interestingly, among grade II patients in the longitudinal sample, those with one or more neurological disabilities reported significantly better physical functioning at baseline compared to those without disabilities. However, a significant interaction with time revealed that their functioning showed a greater decline over time. This unexpected finding may be partly explained by a selection bias, as the majority in our sample had no neurological deficits, limiting our ability to fully understand the impact of neurological disabilities on functioning. In summary, these findings regarding age and neurological disabilities suggest that factors typically associated with functioning or fitness in the general population may not be necessarily relevant in glioma patients, highlighting the importance of broadening our perspective on functioning and fitness in glioma patients.

Additionally, among grade II patients in the longitudinal sample, those diagnosed with an oligodendroglioma reported lower fitness than those with an astrocytoma at baseline. This may be explained by the typically slow-growing nature of oligodendrogliomas, which results in prolonged symptoms before diagnosis.^35^ However, a significant interaction between time and histology indicated that fitness improved more over time in patients with an oligodendroglioma. This may reflect the higher potential for recovery in this subgroup, possibly due to favorable prognostic features.^35^

This study unfortunately did not include patients’ objectively or subjectively assessed level of physical activity. In general, physical activity is crucial for maintaining or improving overall functioning and fitness.^36^ Previous studies found that most brain tumor patients do not meet recommended activity levels from diagnosis through follow-up after treatment, with less activity during or after treatment compared to before diagnosis.^37–39^ In a sample of 18 high-grade glioma patients, 75% were physically active for more than 3 hours per week prior to diagnosis, while only 9% maintained the same level of activity at 1-year follow-up.^40^ This may help explain the decrease in functioning observed in our longitudinal sample of grade III patients between the preoperative and after primary treatment timepoints. Future studies focusing on physical functioning and fitness should also assess levels of physical activity to better understand why some patients experience problems with their functioning and fitness. In addition, incorporating objectively measured physical functioning and fitness could provide further insights and complement patient-reported outcomes. Moreover, exercise interventions for glioma patients should be explored in order to improve levels of physical activity and ultimately to improve self-reported functioning and fitness.^6,41–43^

Psychological and neurocognitive complaints are common in glioma patients^5,6,44^ and may relate to both functioning and fitness. These factors possibly affect not only patients’ engagement in physical activity but also their perception of functioning and fitness. Incorporating these outcomes into future research is also important, as it will offer a more comprehensive understanding of the relationship between physical and cognitive health in this population.

A limitation of this study is a possible selection bias: less-affected patients are more likely to complete questionnaires than more severely impaired patients, as is the case for most research in this patient population. This can lead to an overestimation of functioning and fitness scores in glioma patients, particularly in patients with high-grade glioma, as the included patients represent only a subset of this usually more affected population. However, we might expect that the patients who do fill in the questionnaires would also be more likely to participate and benefit from personalized exercise interventions to improve functioning and fitness, thus still being highly relevant for future studies and improvements in treatment. Another limitation of this study is that tumor classification only partially aligns with the current World Health Organization standards due to missing molecular data in older cases. In addition, due to small sample sizes and missing data, it was not feasible to include variables such as type of surgery, preoperative use of dexamethasone, and preoperative epilepsy consistently across all timepoints. The impact of dexamethasone would be particularly interesting for future research. On the one hand, previous studies have reported negative effects of corticosteroids on muscle strength and the development of myopathy, which may adversely affect functioning and fitness.^45,46^ On the other hand, dexamethasone has also been associated with temporary improvements in cancer-related fatigue, which may positively influence functioning and fitness.^47^

In conclusion, self-reported functioning was particularly low in grade III glioma patients compared to the general population, and they experienced a significant decline after primary treatment. While grade II patients reported better outcomes in this study, large individual variability across all grades emphasizes the need for a personalized approach. Future research should further explore differences in functioning and fitness between tumor grades. Taken all together, these results highlight the importance of expanding our perspective and addressing functioning and fitness in glioma patients.

## Supplementary Material

npaf076_Supplementary_Figure_1

npaf076_Supplementary_Table_1

## Data Availability

Data will be made available upon reasonable request. The scripts of the analyses can be openly accessed on our github repository: https://github.com/multinetlab-amsterdam/projects.
