# Supplementary material for: Self-reported physical functioning and physical fitness in glioma patients
Source: Neurooncol Pract. 2025 Jul 31;13(1):149–61. doi: 10.1093/nop/npaf076 (PMC12965659; doi:10.1093/nop/npaf076)
Supplement: npaf076_Supplementary_Figure_1 [file npaf076_supplementary_figure_1.zip › New folder/Figure captions supplementary.docx]

# Supplementary figure captions

**Figure 1.** This figure shows the distinction between physical functioning and physical fitness. Physical functioning refers to the ability to perform activities of daily living. Physical fitness refers to aerobic functioning. In this study, we focused on self-reported outcomes.

**Figure 2.** The range between the preoperative timepoint and surgery for the total sample of glioma patients.

**Figure 3.** The range between the after primary treatment timepoint and surgery for the total sample of glioma patients.
