# Supplementary figures and images for: Self-reported physical functioning and physical fitness in glioma patients
Source: Neurooncol Pract. 2025 Jul 31;13(1):149–61. doi: 10.1093/nop/npaf076 (PMC12965659; doi:10.1093/nop/npaf076)

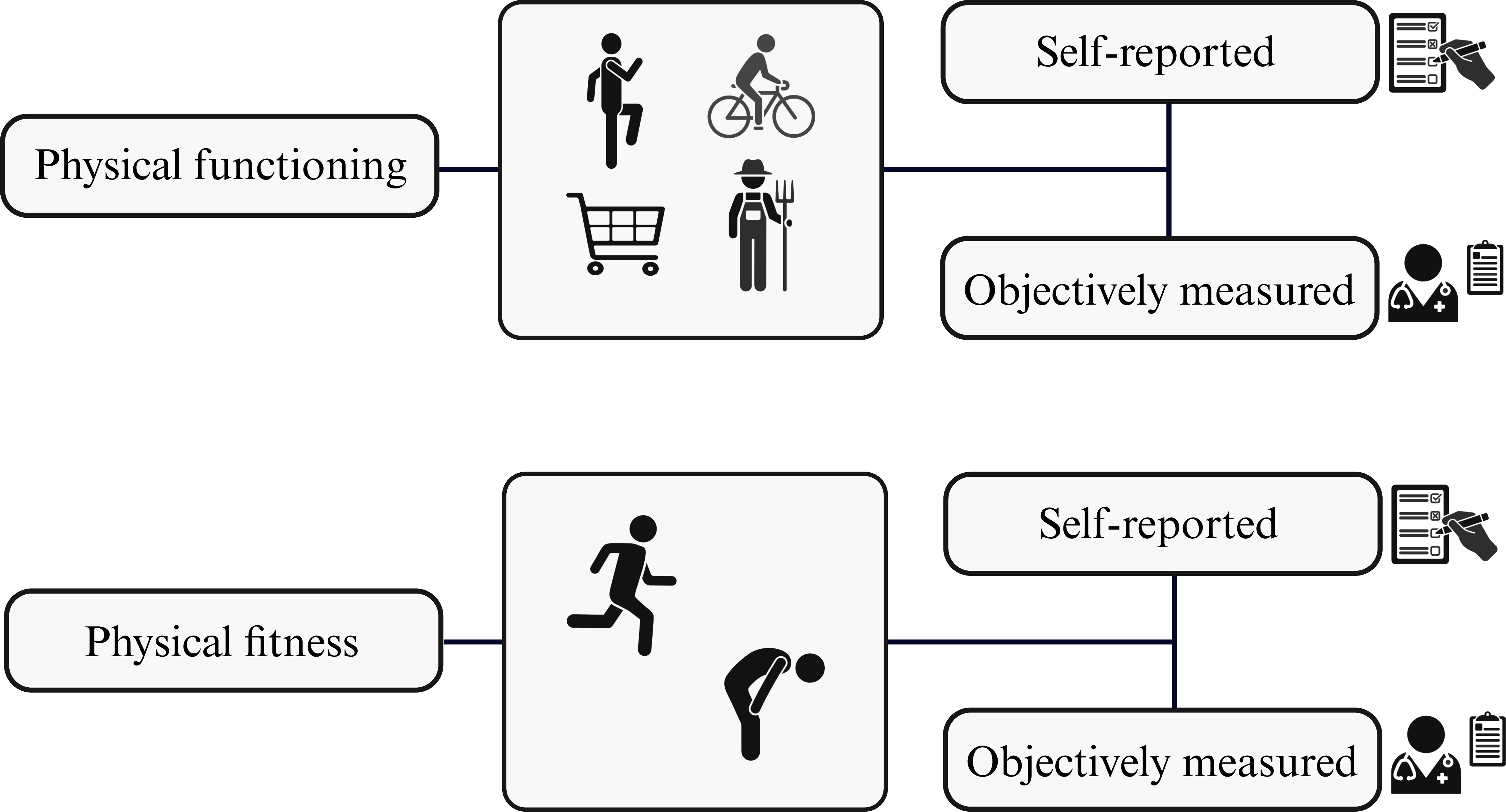

Supplement: npaf076_Supplementary_Figure_1 [file npaf076_supplementary_figure_1.zip › New folder/Supplementary figure 1.tif]

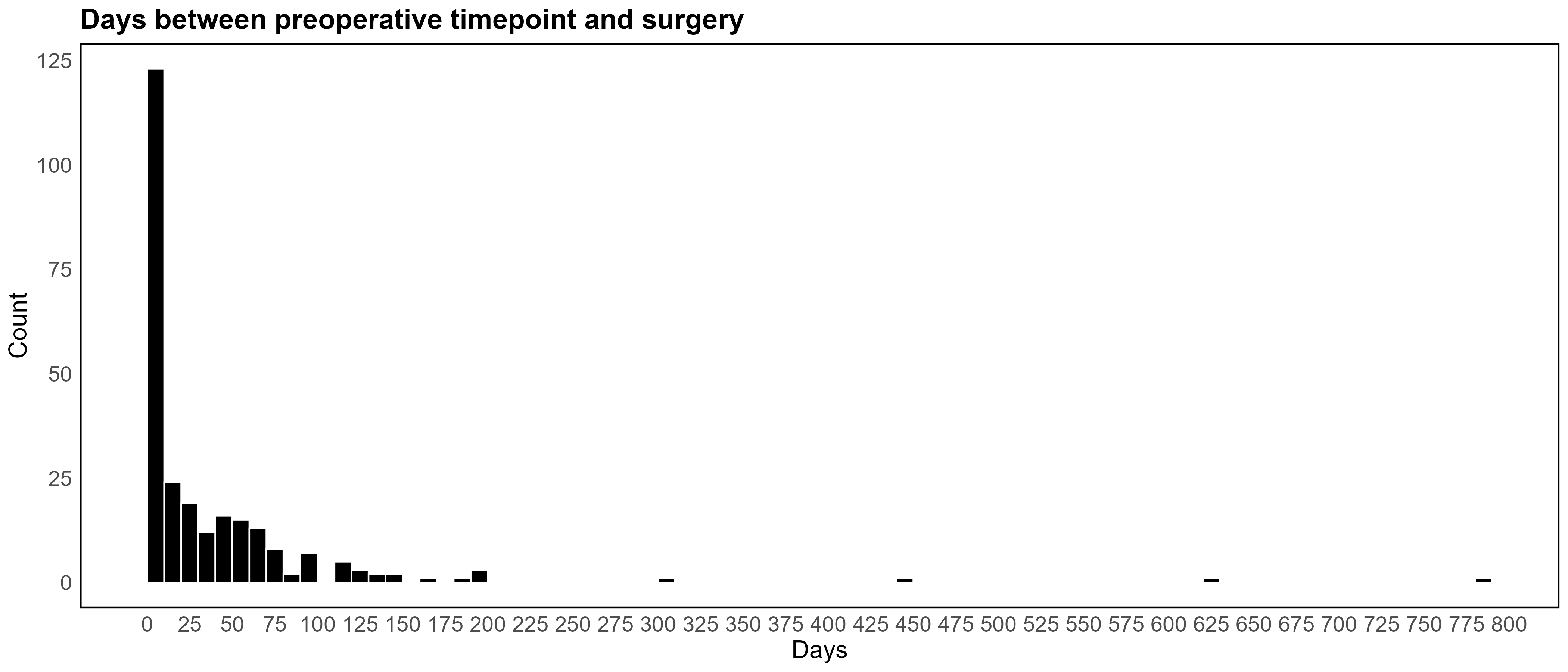

Supplement: npaf076_Supplementary_Figure_1 [file npaf076_supplementary_figure_1.zip › New folder/Supplementary figure 2.tif]

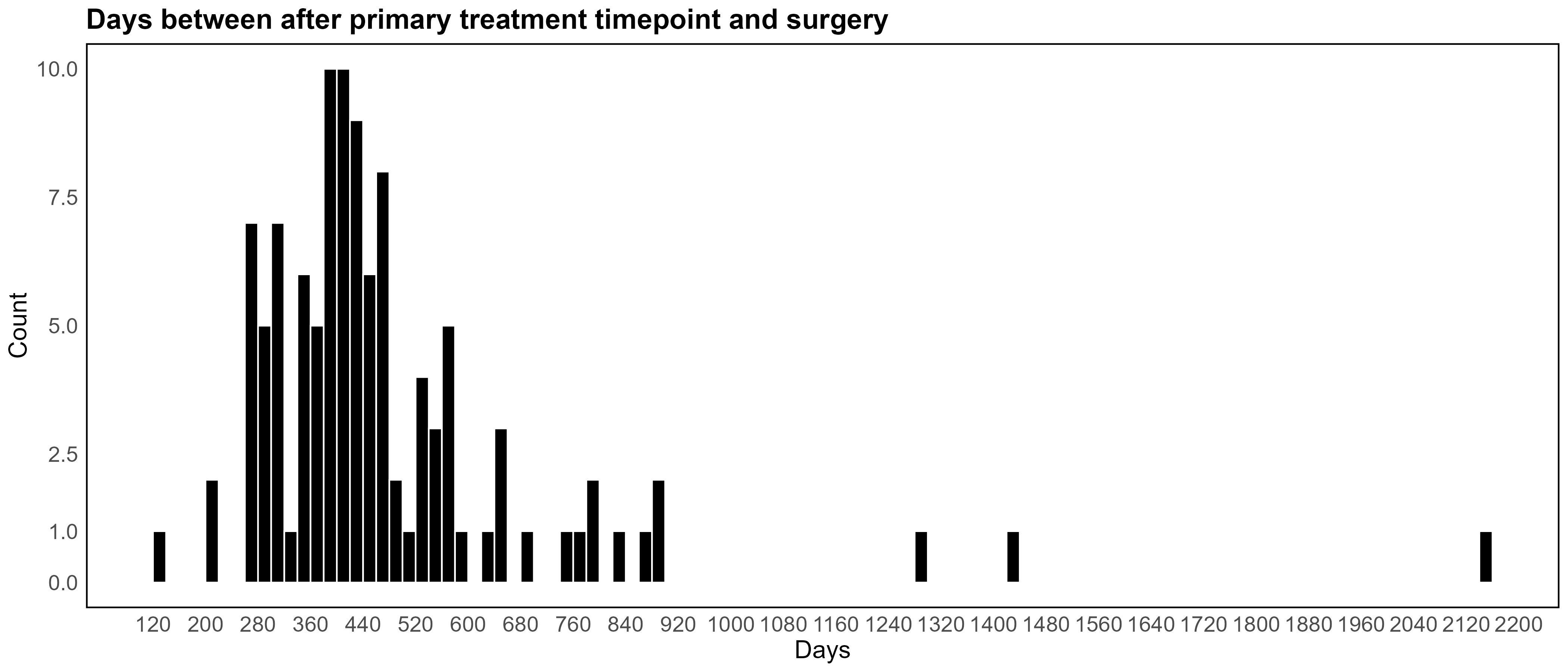

Supplement: npaf076_Supplementary_Figure_1 [file npaf076_supplementary_figure_1.zip › New folder/Supplementary figure 3.tiff]
