# Supplementary material for: Self-reported physical functioning and physical fitness in glioma patients
Source: Neurooncol Pract. 2025 Jul 31;13(1):149–61. doi: 10.1093/nop/npaf076 (PMC12965659; doi:10.1093/nop/npaf076)
Supplement: npaf076_Supplementary_Table_1 [file npaf076_supplementary_table_1.docx]

**Table 1.** Univariate analyses of functioning and fitness preoperatively for grade II, III, and IV glioma patients

|  | **Grade II** | | **Grade III** | | **Grade IV** | |
| --- | --- | --- | --- | --- | --- | --- |
| **SF36 physical functioning** | | | | | | |
|  | Spearman’s rank | | | | | |
|  | ρ | p value | ρ | p value | ρ | p value |
| Age | -0.34 | <0.001*** | -0.13 | 0.320 | -0.03 | 0.810 |
|  | Mann-Whitney U | | | | | |
|  | w value | p value | w value | p value | w value | p value |
| Sex | 1812 | 0.101 | 397.5 | 0.730 | 808 | 0.076 |
| Tumor location | 1667.5 | 0.518 | 298.5 | 0.072 | 968 | 0.280 |
| Tumor lateralization | 1810 | 0.066 | 442.5 | 0.528 | 1023 | 0.031* |
| Histology | 1877.5 | 0.063 | 374 | 0.728 | 230 | 0.542 |
| Epilepsy | 1215.5 | 0.056 | 186.5 | 0.310 | 651.5 | 0.697 |
| Use of dexamethasone | 271.5 | 0.899 | 283 | 0.110 | 895.5 | 0.267 |
| KPS | 94 | <0.001*** | 64.5 | <0.001*** | 62 | <0.001*** |
| NIHSS | 209.5 | 0.859 | 199.5 | 0.123 | 202.5 | 0.491 |
|  | Kruskal-Wallis | | | | | |
|  | H | p value | H | p value | H | p value |
| Educational level | 8.28 | 0.016* | 5.05 | 0.080 | 3.08 | 0.214 |
| **CIS20 physical fitness** | | |  |  |  |  |
|  | Spearman’s rank | | | | | |
|  | ρ | p value | ρ | p value | ρ | p value |
| Age | -0.06 | 0.545 | 0.01 | 0.927 | 0.03 | 0.822 |
|  | Mann-Whitney U | | | | | |
|  | w value | p value | w value | p value | w value | p value |
| Sex | 1835 | 0.098 | 451 | 0.814 | 677 | 0.104 |
| Tumor location | 1666 | 0.503 | 352.5 | 0.268 | 553.5 | 0.064 |
| Tumor lateralization | 1733 | 0.188 | 522.5 | 0.106 | 794 | 0.229 |
| Histology | 1712 | 0.404 | 396 | 0.785 | 145 | 0.449 |
| Epilepsy | 1064.5 | 0.468 | 286.5 | 0.342 | 629 | 0.841 |
| Use of dexamethasone | 229.5 | 0.640 | 230.5 | 0.433 | 750 | 0.710 |
| KPS | 158.5 | 0.004** | 99.5 | <0.001*** | 68.5 | 0.002** |
| NIHSS | 188 | 0.857 | 162.5 | 0.831 | 246 | 0.528 |
|  | Kruskal-Wallis | | | | | |
|  | H | p value | H | p value | H | p value |
| Educational level | 9.40 | 0.009** | 2.87 | 0.237 | 0.29 | 0.865 |

*SF36 = 36-Item Short Form Health Survey; KPS = Karnofsky Performance Score; NIHSS = National Institutes of Health Stroke Scale; CIS20 = Checklist Individual Strength. * < 0.05, ** < 0.01, *** < 0.001.*

**Table 2.** Univariate analyses of functioning and fitness after primary treatment for grade II and III glioma patients

|  | **Grade II** | | **Grade III** | |
| --- | --- | --- | --- | --- |
| **SF36 physical functioning** |  | | | |
|  | Spearman’s rank | | | |
|  | ρ | p value | ρ | p value |
| Age | -0.21 | 0.082 | -0.42 | 0.020* |
|  | Mann-Whitney U | | | |
|  | w value | p value | w value | p value |
| Sex | 623.5 | 0.347 | 173 | 0.036* |
| Tumor location | 645.5 | 0.322 | 137 | 0.431 |
| Tumor lateralization | 604 | 0.492 | 80.5 | 0.228 |
| Histology | 697 | 0.163 | 123 | 0.067 |
| Epilepsy | 423.5 | 0.350 | 43 | 0.973 |
| KPS | 65 | 0.027* | 29.5 | 0.028* |
| NIHSS | 173 | 0.678 | 57 | 0.299 |
|  | Kruskal-Wallis | | | |
|  | H | p value | H | p value |
| Educational level | 3.76 | 0.152 | 1.43 | 0.489 |
| Chemo- and/or radiotherapy | 3.34 | 0.341 | 1.98 | 0.372 |
| **CIS20 physical fitness** | | |  |  |
|  | Spearman’s rank | | | |
|  | ρ | p value | ρ | p value |
| Age | -0.18 | 0.129 | -0.13 | 0.492 |
|  | Mann-Whitney U | | | |
|  | w value | p value | w value | p value |
| Sex | 650.5 | 0.259 | 141.5 | 0.235 |
| Tumor location | 544 | 0.782 | 116 | 0.750 |
| Tumor lateralization | 655.5 | 0.206 | 87 | 0.581 |
| Histology | 608 | 0.738 | 96.5 | 0.445 |
| Epilepsy | 302.5 | 0.348 | 52.5 | 0.425 |
| KPS | 72.5 | 0.0499* | 27.5 | 0.064 |
| NIHSS | 183 | 0.869 | 54.5 | 0.159 |
|  | Kruskal-Wallis | | | |
|  | H | p value | H | p value |
| Educational level | 1.84 | 0.399 | 2.15 | 0.342 |
| Chemo- and/or radiotherapy | 0.69 | 0.875 | 1.10 | 0.578 |

*SF36 = 36-Item Short Form Health Survey; KPS = Karnofsky Performance Score; NIHSS = National Institutes of Health Stroke Scale; CIS20 = Checklist Individual Strength. * < 0.05.*

**Table 3.** Univariate analyses of the longitudinal change in functioning and fitness in grade II glioma patients

|  | **SF36**  **physical functioning** | | **CIS20**  **physical fitness** | |
| --- | --- | --- | --- | --- |
|  | t value | p value | t value | p value |
| Age | -0.22 | 0.825 | 0.51 | 0.611 |
| Sex | -0.83 | 0.408 | -0.77 | 0.443 |
| Educational level middle compared to low | -0.11 | 0.911 | 0.67 | 0.509 |
| Educational level high compared to low | 0.59 | 0.561 | 2.00 | 0.052 |
| Histology oligodendroglioma compared to astrocytoma | -1.66 | 0.105 | -2.52 | 0.015* |
| Tumor location non frontal compare to frontal | 0.43 | 0.667 | -2.19 | 0.034* |
| Tumor lateralization right compared to left | -1.18 | 0.245 | -0.23 | 0.822 |
| Epilepsy | -0.46 | 0.648 | -1.07 | 0.289 |
| KPS ≥ 80 compared to ≤ 70 | 0.63 | 0.531 | 0.98 | 0.333 |
| NIHSS one or more disabilities compared to none | 2.36 | 0.024* | 0.39 | 0.699 |
| Only radiotherapy compared to none | 0.54 | 0.595 | 0.97 | 0.338 |
| Only chemotherapy compared to none | 1.34 | 0.189 | 1.11 | 0.273 |
| Both chemo- and radiotherapy compared to none | 0.84 | 0.404 | 0.70 | 0.489 |
|  |  |  |  |  |
| Time x age | -1.16 | 0.250 | -1.59 | 0.118 |
| Time x sex | 0.32 | 0.747 | 0.09 | 0.929 |
| Time x educational level middle | 0.40 | 0.694 | -0.85 | 0.398 |
| Time x educational level high | -0.03 | 0.977 | -1.67 | 0.101 |
| Time x oligodendroglioma | 0.92 | 0.364 | 2.03 | 0.049* |
| Time x tumor location non frontal | -1.06 | 0.295 | 1.85 | 0.070 |
| Time x tumor lateralization right | 0.57 | 0.566 | -0.54 | 0.589 |
| Time x epilepsy | 0.13 | 0.900 | 1.33 | 0.192 |
| Time x KPS ≥ 80 | -0.17 | 0.867 | -0.35 | 0.727 |
| Time x NIHSS one or more disabilities | -2.27 | 0.030* | -0.53 | 0.601 |
| Time x only radiotherapy | -0.55 | 0.586 | -0.39 | 0.697 |
| Time x only chemotherapy | -0.77 | 0.448 | -0.68 | 0.499 |
| Time x both chemo- and radiotherapy | -0.64 | 0.524 | -0.56 | 0.579 |

*SF36 = 36-Item Short Form Health Survey; CIS20 = Checklist Individual Strength; KPS = Karnofsky Performance Score; NIHSS = National Institutes of Health Stroke Scale. * < 0.05.*

**Table 4.** Univariate analyses of the longitudinal change in functioning and fitness in grade III glioma patients

|  | **SF36**  **physical functioning** | | **CIS20**  **physical fitness** | |
| --- | --- | --- | --- | --- |
|  | t value | p value | t value | p value |
| Age | 1.11 | 0.281 | 0.20 | 0.841 |
| Sex | 1.50 | 0.150 | -0.02 | 0.987 |
| Educational level high compared to middle | 0.99 | 0.334 | -1.67 | 0.112 |
| Histology oligodendroglioma compared to astrocytoma | 0.64 | 0.528 | 0.92 | 0.372 |
| Tumor location non frontal compare to frontal | 1.23 | 0.234 | 0.36 | 0.724 |
| Tumor lateralization right compared to left | 0.57 | 0.577 | -2.06 | 0.054 |
| KPS ≥ 80 compared to ≤ 70 | 1.18 | 0.254 | 1.01 | 0.328 |
| NIHSS one or more disabilities compared to none | -0.98 | 0.349 | -0.35 | 0.731 |
| Only radiotherapy compared to none | -1.09 | 0.293 | 1.25 | 0.228 |
| Both chemo- and radiotherapy compared to none | 0.84 | 0.415 | 1.83 | 0.086 |
|  |  |  |  |  |
| Time x age | -2.00 | 0.060 | -0.30 | 0.765 |
| Time x sex | -1.95 | 0.067 | -0.48 | 0.634 |
| Time x educational level high | -0.60 | 0.553 | 1.86 | 0.079 |
| Time x oligodendroglioma | -0.86 | 0.399 | -0.94 | 0.362 |
| Time x tumor location non frontal | -1.50 | 0.153 | -0.61 | 0.549 |
| Time x tumor lateralization right | 0.27 | 0.790 | 2.27 | 0.036* |
| Time x KPS ≥ 80 | -0.17 | 0.866 | -0.50 | 0.622 |
| Time x NIHSS one or more disabilities | 0.42 | 0.686 | -0.17 | 0.868 |
| Time x only radiotherapy | 1.09 | 0.290 | -0.85 | 0.408 |
| Time x both chemo- and radiotherapy | -1.39 | 0.183 | -2.14 | 0.048* |

*SF36 = 36-Item Short Form Health Survey; CIS20 = Checklist Individual Strength; KPS = Karnofsky Performance Score; NIHSS = National Institutes of Health Stroke Scale. * < 0.05.*

**Table 5.** Multivariate analyses of the longitudinal change in functioning and fitness in grade II glioma patients

|  | Estimate | Std. Error | DF | t value | p value |
| --- | --- | --- | --- | --- | --- |
| **SF36 physical functioning** |  |  |  |  |  |
| (Intercept) | 97.9 | 24.6 | 33 | 3.98 | <0.001* |
| Time | -2.78 | 10.2 | 30 | -0.27 | 0.787 |
| Age | -0.33 | 0.46 | 33 | -0.72 | 0.478 |
| Sex | 1.21 | 9.37 | 33 | 0.13 | 0.898 |
| Educational level middle compared to low | -13.3 | 15.3 | 33 | -0.87 | 0.389 |
| Educational level high compared to low | 2.28 | 14.1 | 33 | 0.16 | 0.872 |
| NIHSS one or more disabilities compared to none | 33.4 | 13.6 | 30 | 2.46 | 0.020* |
|  |  |  |  |  |  |
| Time x age | 0.16 | 0.19 | 30 | 0.84 | 0.410 |
| Time x sex | -2.03 | 3.89 | 30 | -0.52 | 0.606 |
| Time x educational level middle | 6.98 | 6.30 | 30 | 1.11 | 0.277 |
| Time x educational level high | -0.31 | 5.84 | 30 | -0.05 | 0.959 |
| Time x NIHSS one or more disabilities | -16.3 | 6.75 | 30 | -2.41 | 0.022* |
| **CIS20 physical fitness** |  |  |  |  |  |
| (Intercept) | 8.85 | 7.02 | 40 | 1.26 | 0.215 |
| Time | 7.38 | 4.00 | 40 | 1.84 | 0.073 |
| Age | 0.15 | 0.12 | 40 | 1.25 | 0.220 |
| Sex | -3.62 | 2.54 | 40 | -1.43 | 0.161 |
| Educational level middle compared to low | 4.68 | 4.23 | 40 | 1.10 | 0.276 |
| Educational level high compared to low | 6.97 | 4.09 | 40 | 1.70 | 0.096 |
| Histology oligodendroglioma compared to astrocytoma | -7.02 | 2.70 | 40 | -2.60 | 0.013* |
| Tumor location non frontal compare to frontal | -3.09 | 2.86 | 40 | -1.08 | 0.287 |
|  |  |  |  |  |  |
| Time x age | -0.17 | 0.07 | 40 | -2.48 | 0.018* |
| Time x sex | 1.04 | 1.45 | 40 | 0.72 | 0.477 |
| Time x educational level middle | -4.09 | 2.41 | 40 | -1.69 | 0.098 |
| Time x educational level high | -3.88 | 2.33 | 40 | -1.66 | 0.104 |
| Time x oligodendroglioma | 3.84 | 1.54 | 40 | 2.49 | 0.017* |
| Time x tumor location non frontal | 1.46 | 1.63 | 40 | 0.89 | 0.377 |

*SF36 = 36-Item Short Form Health Survey; KPS = Karnofsky Performance Score; NIHSS = National Institutes of Health Stroke Scale; CIS20 = Checklist Individual Strength. * < 0.05.*

**Table 6.** Multivariate analyses of the longitudinal change in functioning and fitness in grade III glioma patients

|  | Estimate | Std. Error | DF | t value | p value |
| --- | --- | --- | --- | --- | --- |
| **SF36 physical functioning** |  |  |  |  |  |
| (Intercept) | 43.9 | 29.8 | 16 | 1.47 | 0.160 |
| Time | 36.3 | 17.8 | 16 | 2.04 | 0.058 |
| Age | 0.77 | 0.63 | 16 | 1.23 | 0.238 |
| Sex | 22.6 | 14.2 | 16 | 1.59 | 0.132 |
| Educational level high compared to middle | 22.3 | 14.4 | 16 | 1.55 | 0.141 |
|  |  |  |  |  |  |
| Time x age | -0.80 | 0.38 | 16 | -2.13 | 0.049* |
| Time x sex | -17.2 | 8.54 | 16 | -2.01 | 0.061 |
| Time x educational level high | -12.8 | 8.63 | 16 | -1.48 | 0.158 |
| **CIS20 physical fitness** |  |  |  |  |  |
| (Intercept) | 19.3 | 11.8 | 13 | 1.64 | 0.125 |
| Time | -3.97 | 6.78 | 13 | -0.59 | 0.569 |
| Age | -0.16 | 0.22 | 13 | -0.72 | 0.486 |
| Sex | -2.13 | 5.14 | 13 | -0.414 | 0.686 |
| Educational level high compared to middle | -4.24 | 4.98 | 13 | -0.85 | 0.410 |
| Tumor lateralization right compared to left | -7.40 | 5.70 | 13 | -1.30 | 0.216 |
| Only radiotherapy compared to none | 5.08 | 10.8 | 13 | 0.47 | 0.645 |
| Both chemo- and radiotherapy compared to none | 9.39 | 6.94 | 13 | 1.35 | 0.199 |
|  |  |  |  |  |  |
| Time x age | 0.12 | 0.13 | 13 | 0.90 | 0.385 |
| Time x sex | 0.59 | 2.96 | 13 | 0.20 | 0.846 |
| Time x educational level high | 2.53 | 2.87 | 13 | 0.88 | 0.394 |
| Time x tumor lateralization right compared to left | 5.41 | 3.28 | 13 | 1.65 | 0.123 |
| Time x only radiotherapy compared to none | -0.83 | 6.19 | 13 | -0.13 | 0.895 |
| Time x both chemo- and radiotherapy | -6.82 | 4.00 | 13 | -1.71 | 0.112 |

*SF36 = 36-Item Short Form Health Survey; KPS = Karnofsky Performance Score; NIHSS = National Institutes of Health Stroke Scale; CIS20 = Checklist Individual Strength. * < 0.05.*

**Table 7.** Functioning and fitness of grade II, III, and IV glioma patients compared to controls within narrower time ranges

|  | **N** | **Group** | **Median (IQR)** | **U** | **Z** | **p-value** |
| --- | --- | --- | --- | --- | --- | --- |
| **Preoperatively** |  |  |  |  |  |  |
| **SF36 physical functioning** |  |  |  |  |  |  |
| Grade II | 94 | Patients | 95 (90-100) | 8310 | -0.81 | 0.393 |
|  | 188 | Controls | 95 (88-100) |  |  |  |
| Grade III | 54 | Patients | 90 (65-95) | 2354 | -2.00 | 0.042* |
|  | 108 | Controls | 95 (75-100) |  |  |  |
| Grade IV | 82 | Patients | 90 (75-95) | 5810.5 | -1.74 | 0.078 |
|  | 164 | Controls | 90 (80-100) |  |  |  |
| **CIS20 physical fitness** |  |  |  |  |  |  |
| Grade II | 96 | Patients | 15 (9-19) | 8576.5 | -0.96 | 0.336 |
|  | 192 | Controls | 15 (11-18) |  |  |  |
| Grade III | 55 | Patients | 12 (7-16.5) | 2470.5 | -1.92 | 0.055 |
|  | 110 | Controls | 14 (11-19) |  |  |  |
| Grade IV | 76 | Patients | 14 (10-19) | 5227.5 | -1.17 | 0.242 |
|  | 152 | Controls | 16 (11-18.25) |  |  |  |
| **After primary treatment** |  |  |  |  |  |  |
| **SF36 physical functioning** |  |  |  |  |  |  |
| Grade II | 49 | Patients | 95 (85-100) | 2172.5 | -0.94 | 0.326 |
|  | 98 | Controls | 95 (90-100) |  |  |  |
| Grade III | 21 | Patients | 75 (60-95) | 259.5 | -2.65 | 0.006** |
|  | 42 | Controls | 95 (90-100) |  |  |  |
| Grade IV | 5 | Patients | 100 (60-100) | 29 | 0.49 | 0.661 |
|  | 10 | Controls | 90 (76.25-93.75) |  |  |  |
| **CIS20 physical fitness** |  |  |  |  |  |  |
| Grade II | 49 | Patients | 14 (9-18) | 2218.5 | -0.75 | 0.453 |
|  | 98 | Controls | 14 (11-18) |  |  |  |
| Grade III | 20 | Patients | 13.5 (8.75-16.25) | 322 | -1.22 | 0.223 |
|  | 40 | Controls | 16 (10.75-18.25) |  |  |  |
| Grade IV | 5 | Patients | 17 (14-18) | 18 | -0.86 | 0.417 |
|  | 10 | Controls | 19 (16.25-21) |  |  |  |

*SF36 = 36-Item Short Form Health Survey CIS20 = Checklist Individual Strength. * < 0.05; ** <0.01.*
